# Enhanced gene delivery in tumor cells using chemical carriers and mechanical loadings

**DOI:** 10.1371/journal.pone.0209199

**Published:** 2018-12-28

**Authors:** Amin Hadi, Abbas Rastgoo, Nooshin Haghighipour, Azam Bolhassani, Fatemeh Asgari, Sepehr Soleymani

**Affiliations:** 1 School of Mechanical Engineering, University of Tehran, Tehran, Iran; 2 National Cell Bank of Iran, Pasteur Institute of Iran, Tehran, Iran; 3 Department of Hepatitis and AIDs, Pasteur Institute of Iran, Tehran, Iran; University of Zaragoza, SPAIN

## Abstract

Intracellular delivery of DNA is considered a challenge in biological research and treatment of diseases. The previously reported transfection rate by commercially available transfection reagents in cancer cell lines, such as the mouse lung tumor cell line (TC-1), is very low. The purpose of this study is to introduce and optimize an efficient gene transfection method by mechanical approaches. The combinatory transfection effect of mechanical treatments and conventional chemical carriers is also investigated on a formerly reported hard-to-transfect cell line (TC-1). To study the effect of mechanical loadings on transfection rate, TC-1 tumor cells are subjected to uniaxial cyclic stretch, equiaxial cyclic stretch, and shear stress. The TurboFect transfection reagent is exerted for chemical transfection purposes. The pEGFP-N1 vector encoding the green fluorescent protein (GFP) expression is utilized to determine gene delivery into the cells. The results show a significant DNA delivery rate (by ~30%) in mechanically transfected cells compared to the samples that were transfected with chemical carriers. Moreover, the simultaneous treatment of TC-1 tumor cells with chemical carriers and mechanical loadings significantly increases the gene transfection rate up to ~ 63% after 24 h post-transfection. Our results suggest that the simultaneous use of mechanical loading and chemical reagent can be a promising approach in delivering cargoes into cells with low transfection potentials and lead to efficient cancer treatments.

## Introduction

Managing the transportation of molecules in biological cells is a significant aim in many medical processes including gene therapy and treatment of diseases such as cancer and viral diseases [1]. From the various DNA transfection methods applied for eukaryotic cells, some methods rely on physical treatments and others rely on chemical materials or biological particles as the functional carriers. Chemical carrier can have a polymeric (such as polyplexes) or a lipid base (such as lipofection) [1]. Transfection using physical methods, such as electroporation and sonoporation, is challenging as they showed to physically disrupt the cell membrane [2]. Many researchers have developed several physical methods for gene transfection [3–5].

Physical methods suggested for transfection have shown an advantage in some applications. These methods eliminate the need for vector materials and circumvent the endocytotic pathway; especially those involving primary cells that are recalcitrant to vector-based methods. However, both electroporation and sonoporation techniques have the disadvantage of being highly toxic and have shown limited success in delivering materials such as proteins and nano-materials. Electroporation, in particular, has been shown to damage certain target materials, such as quantum dots [6, 7]. Moreover, electroporation has a harmful effect on cells, which is related to its effect on pH levels. [8]. Microinjection, an alternative method in which cells are punctured by a microneedle, can address a variety of target materials and cell types. However, its low throughput has hindered its adoption for most applications (throughput∼100 cell/h at most) [9]. Thus, there is need for more effective intracellular delivery methods.

Mechanotransduction is a process where cells transmute mechanical stimuli into electrochemical signals. Mechanotransduction plays a momentous role in many microbiological phenomena such as regenerative medicine [10–12], cell proliferation [13–16], and differentiation [17–23]. However, the exact mechanisms by which cells sense and respond to local mechanical signals are not well understood [24–27]. Mechanotransduction occurs in living cells by various stimuli such as hydrostatic pressure [28–31], cyclic stretch [32–35], and cyclic shear stress [36–38].

The cell membrane is the primary barrier to the transport of molecules and ions between the interior and the exterior regions of a cell [39]. Leontiadou *et al*. [40] presented atomistic MD simulations to show that hydrophilic water pores could be stabilized in a DPPC lipid membrane under low tension. The pores were shaped like an hourglass, with a radius of 0.7 nm in the interior of the membrane [40]. Therefore, we can expect that mechanical stress alone can be a potent transfection factor.

According to the research literature, mechanical stresses exerted on cells can lead to gene transfection. Accordingly, in this paper, three mechanical bioreactors (uniaxial cyclic stretch, equiaxial cyclic stretch, and shear stress) were used to create a different force field on TC-1 tumor cell line for gene transfection (*i*.*e*., pEGFP-N1). The transfection efficiency of the chemical method in combination to mechanical approaches was conducted and compared with the results obtained from mechanical loadings or chemical carrier, alone. In the end, the optimal conditions for gene transfection were determined.

## Materials and methods

### 2.1 Cell line and plasmid DNA construct

The cell line used in this study is TC-1 (NCBI Code: C569) that was provided by the National Cell Bank of Iran (Pasteur Institute of Iran). TC-1 cell line was derived from the primary lung epithelial cells of C57BL/6 mice. The TC-1 lung metastasis model can be used to test the efficacy of various E6/E7-specific vaccines and immunotherapeutic strategies [41]. The pEGFP-N1 plasmid (Clontech, Mountain View, CA) was used for the *in vitro* transfection assay. This vector encoding the GFP marker was purified using the EndoFree Plasmid Maxi Kit (Qiagen, Hilden, Germany) according to the manufacturer’s instructions. DNA concentration was determined using NanoDrop spectrophotometer (Thermo Fisher Scientific Inc., N-1000, USA).

### 2.2 Cell culture

The TC-1 cancerous cell line was cultured in RPMI 1640 (Sigma, Germany) supplemented with 5% heat-inactivated fetal calf serum (Gibco, USA), 2 mM L-glutamine (Sigma, Germany), 5 ×10^−5^ mM 2-mercaptoethanol (Sigma, Germany), 10 mM HEPES (Sigma, Germany), and 40 μg/ml gentamicin (Sigma, Germany). Cells were incubated in a humidified atmosphere at 37°C in 5% CO_2_. TC-1 cells were harvested by trypsinization, counted, and seeded in a 24-well plate and also on a medical grade silicone membrane.

### 2.3 Transfection method by TurboFect reagent

For transfection using the cationic polymer technique, cells were transfected with TurboFect transfection reagent (Thermo Scientific) according to the manufacturer’s protocol. Briefly, the TC-1 cells were seeded at a density of 6 × 10^4^ cells/well in a 24-well plate in complete RPMI-1640 supplemented with 5% heat-inactivated FBS. For DNA transfection with TurboFect, the purified pEGFP-N1 vector (~1 μg) was pre-incubated with 4 μl of reagent in a final volume of 25 μl and incubated at room temperature for 20 min to form the DNA/TurboFect complex. The complex was then added to the cells in each well containing serum-free medium. The medium was replaced after six h incubation at 37°C with complete RPMI 5% FBS. The transfection efficiency was monitored by fluorescence microscopy (EnvertFluorescent Ceti, Korea) and quantified by a FACS Calibur flow cytometer (Partec, Germany) at 24 h post-transfection. Flow cytometer settings were adjusted to discriminate the transfected and non-transfected cells. The Windows FloMax software package was used for data analysis. Moreover, for the simultaneous treatment using the chemical reagent and mechanical loading, cells cultured on the silicone membranes were loaded by various mechanical bioreactors before transfection. Then, the plasmid DNA alone or complexed with TurboFect were added to the medium and the cells were incubated at 37°C in a 5% CO_2_ incubator.

### 2.4 Transfection method by mechanical loading

**Fig 1:**
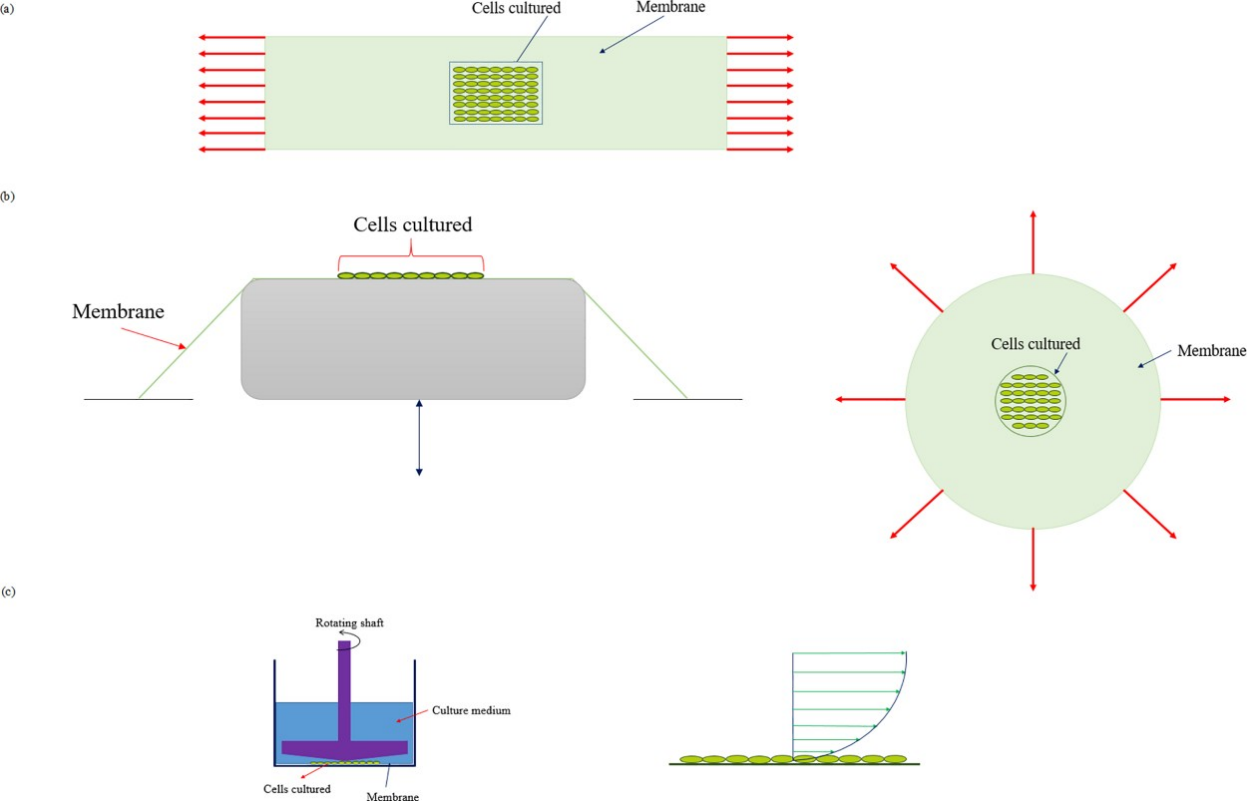
The schematic of three bioreactors. (a) Uniaxial cyclic stretch bioreactor (b) Equiaxial cyclic stretch bioreactor (c) Shear stress bioreactor.

After autoclave sterilization, the medical grade silicone membrane was used as the cell seeding platform for further cell transfection analyses. Briefly, the TC-1 cells were seeded on the central part of the silicone membrane at a density of 2×10^5^ cells and incubated for 2 to 3 hours. After ensured cell attachment to the membrane, by microscopic observations, the culture medium was slowly added to the petri dish and incubated overnight in the incubator. According to the research on molecular dynamics [40], which indicated that mechanical stress was the cause of forming pores on cell wall, it can be expected that the mechanical loadings cause molecules to diffuse into the cell. Therefore, this study focused on the effect of mechanical loading on the cell by various mechanical bioreactors. For applying mechanical loadings on the studied cell line, uniaxial cyclic stretch, equiaxial cyclic stretch and shear stress bioreactors made in the Pasteur Institute of Iran were used. The schematic of these bioreactors were shown in Fig 1. After unloading the cultured cells, the transfection process was further carried out in 2 situations. In one case, the loaded cultured cells were placed in the vicinity of the plasmid DNA containing GFP and without using TurboFect transfection reagent. In this case, the effect of mechanical loading on transfection rate was examined. In the second situation, the loaded cultured cells were placed in the vicinity of the plasmid DNA containing GFP and TurboFect transfection reagent.

#### 2.4.1 Transfection using uniaxial cyclic stretch

Various designs that apply uniaxial cyclic strain to cells cultured on a suitable elastic membrane or soft tissue and simulation of the internal mechanical environment of the body have been investigated, but each design has its limitations. A uniaxial cyclic stretch bioreactor in National Cell Bank of Iran had been designed to apply uniaxial cyclic stretch. In this step, mechanical loading was be used. The Range of applied strain was 0 to 10 percent, at a time interval of 24 hours and a frequency of 0.5HZ. It should be noted that the test conditions must first be determined for any mechanical load to achieve the best transfection efficiency without causing any damage to the cell. After unloading and separating the membrane from the uniaxial cyclic stretch bioreactor, the membrane containing the loaded cell, the transfection operation should be performed according to section 2.3. Flow cytometry and fluorescence microscopy were used for analyzing protein expression in the cell.

#### 2.4.2 Transfection method by equiaxial cyclic stretch bioreactor

In this section, the equiaxial cyclic stretch acted to the cell adhering to the silicone membrane is defined. This bioreactor was able to stretch cyclically into the periphery of a circular membrane. To conduct the test, 2×10^5^ cells were cultured on the center of the membrane and incubated overnight. This test was done at the frequency of 0.5Hz, loading time of 15 min, and 5% strain. After unloading, the membrane was separated from the bioreactor, and the transfection operation was performed as described in section 2.3.

#### 2.4.3 Transfection method by shear stress

The shear stress bioreactor was used in this section for transfection of TC-1. This bioreactor can apply shear stress to the cultured cells on the silicone membrane by a rotating shaft with the frequency *f*, shaft radius *R*, and with a distance of *h* from the membrane. In this bioreactor, the slope of cone clinging to the rotating shaft (0.5–3 degree) is low. For this slope, the flow is laminar. In this case, in the center of the membrane for a small radius, shear stress is almost constant [42]. The applied shear stress was obtained according to the following equation:

$$\tau =\frac{2\pi \mu Rf}{h}$$ (1)

About 2×10^5^ cells were cultured on a center of the silicone membrane and loaded after 24 hours. The cultured cells located at the center of the bioreactor would be exposed to the same shear stress conditions. After unloading, the membrane was separated from the bioreactor and the transfection operation was performed as described in section 2.3.

### 2.5 Statistical analysis

The results are expressed as mean ± standard deviation. The normality test was done by Shapiro-Wilk method. ANOVA and Scheffe's tests (SPSS) were performed to analyze the percentage of transfection using flow cytometry. The value of *p* < 0.05 was considered statistically significant.

## Results

### 3.1 Effect of cationic polymer TurboFect transfection reagent

The pEGFP-N1 transfection in TC1 cells was investigated using TurboFect transfection reagent. Flow cytometry analysis showed that the transfection rate of pEGFP-N1 (*i*.*e*., the percentage of GFP expression) into TC-1 cell is very low (~ 5%) by this method.

### 3.2 Effect of uniaxial cyclic stretch

**Fig 2:**
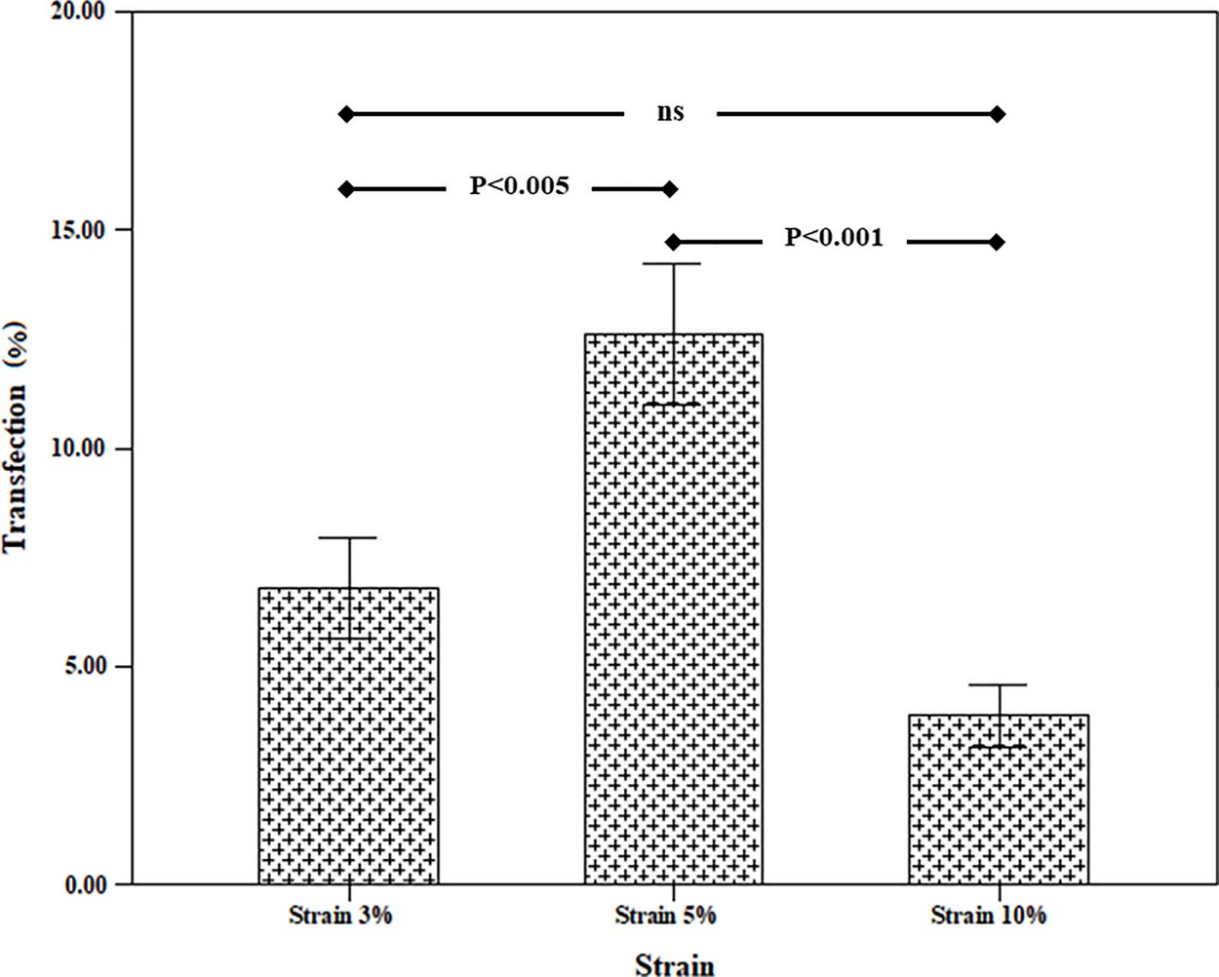
Effect of strain rate on transfection efficiency (loading time = 60 min, frequency = 0.5 Hz).

After uniaxial cyclic stretch transfection, the cells were trypsinized from the medical silicone membrane, and then, the flow cytometry was used for analyzing protein expression in the cells. First, the effect of strain rate was investigated in three strain rates (3%, 5%, and 10%). For this test, the loading time and frequency were 60 minute and 0.5Hz. The results showed the maximum rate of transfection was related to 5% strain (see Fig 2). In the 10% strain, the transfection rate was reduced, which can be due to damage to the cells. The results indicated that there was a significant difference between different strain groups. The statistical test demonstrated that the 5% strain group possessed the most significant differences compared to other related groups.

**Fig 3:**
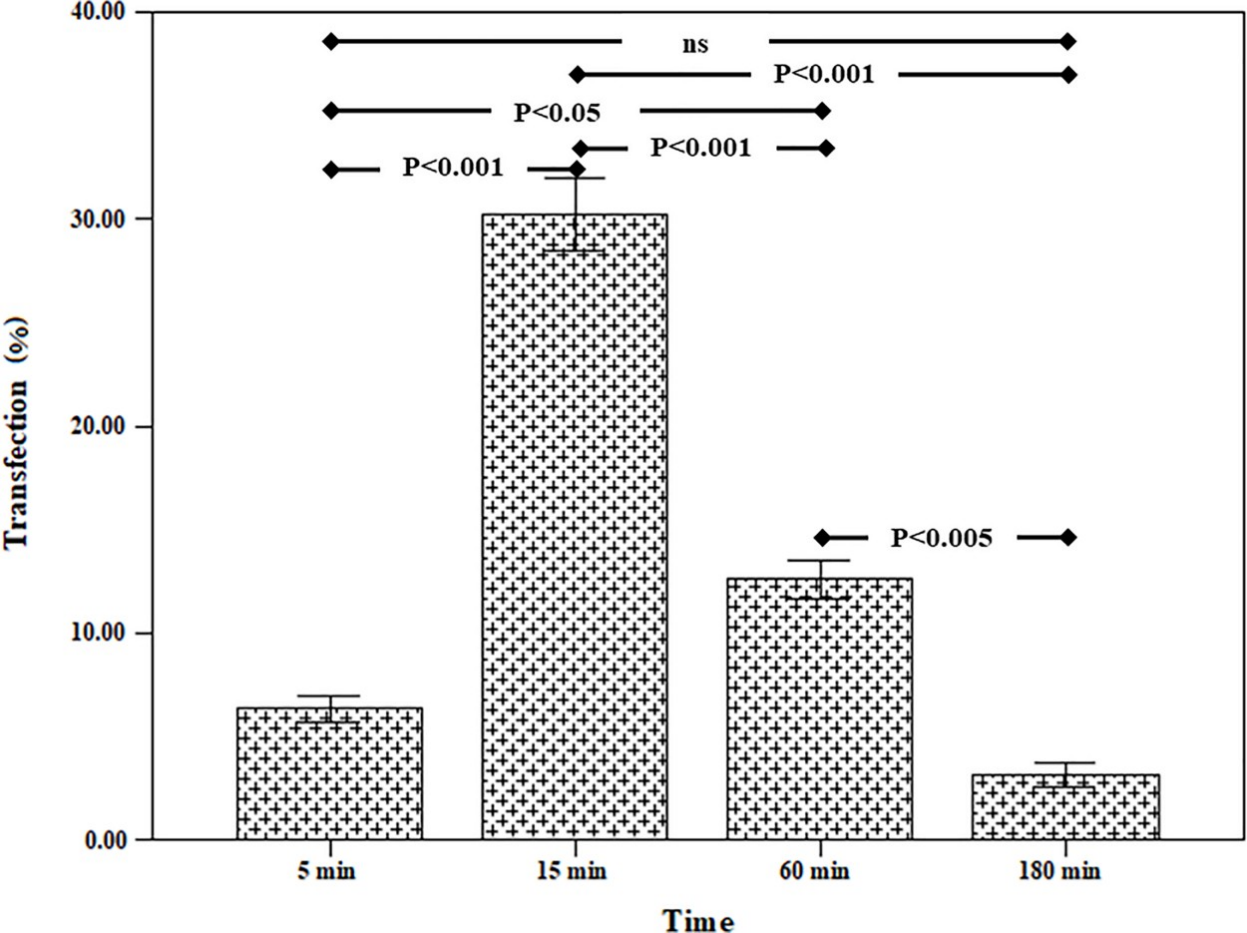
Effect of various loading time on transfection efficiency (5% strain, frequency = 0.5 Hz).

The results for the 5% strain are given in Fig 3 for various loading times. This figure showed that by decreasing the loading time, the transfection rate was increased. The maximum transfection rate was related to the loading time of 15 minutes. At the loading time of 15 minutes, the percentage of transfection was 30.20 ± 3.02%. At the loading time of 5 minutes, the percentage of transfection was reduced by 6.35 ± 1.31%. The statistical test demonstrated that the 5% strain group and 15 minutes of loading possessed the most significant differences compared to other related groups.

### 3.3 The simultaneous use of uniaxial cyclic stretch and TurboFect transfection reagent

**Fig 4:**
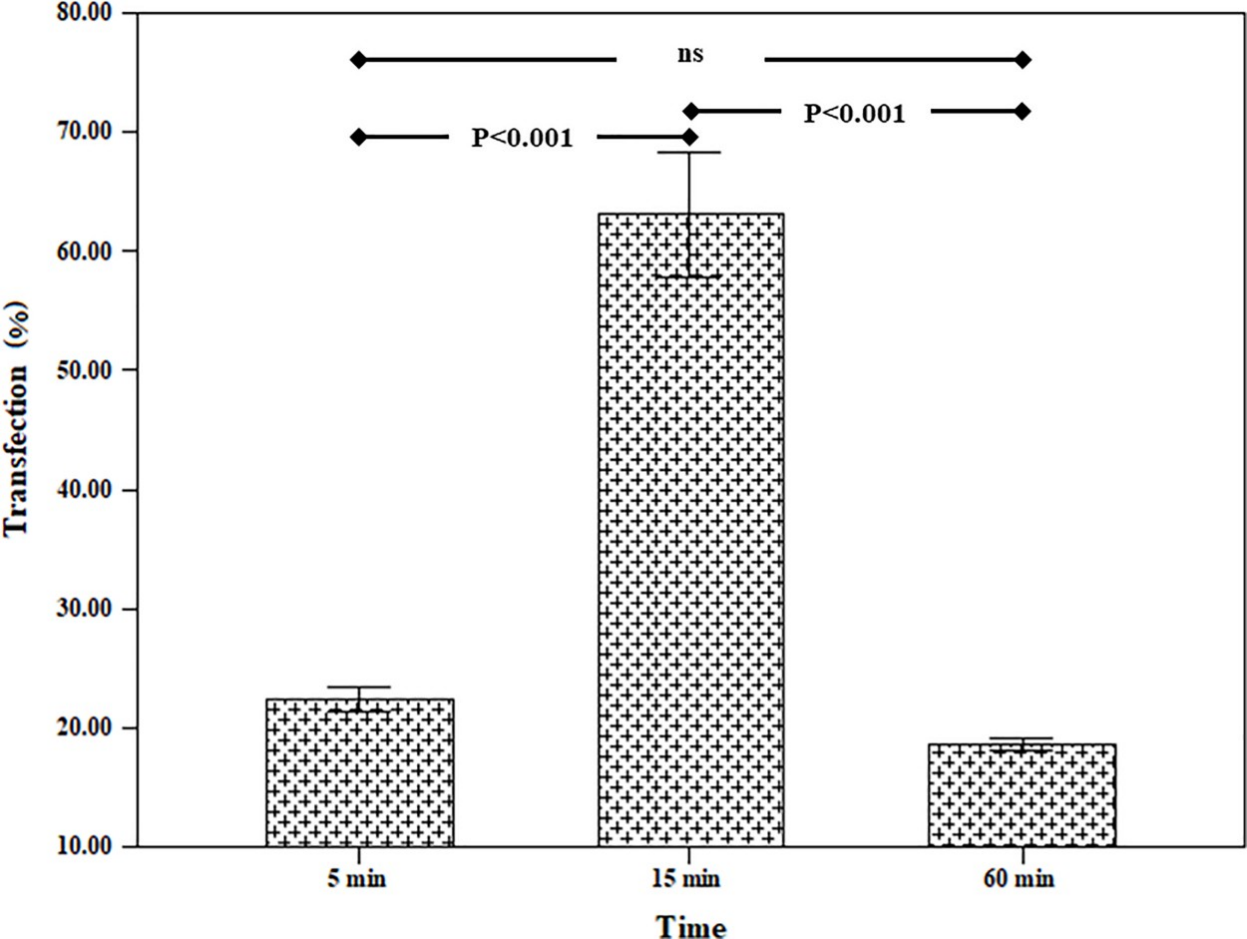
Effect of simultaneous use of uniaxial cyclic stretch and chemical reagent (TurboFect transfection reagent) for various loading time (strain rate = 0.5%, frequency = 0.5 Hz).

**Fig 5:**
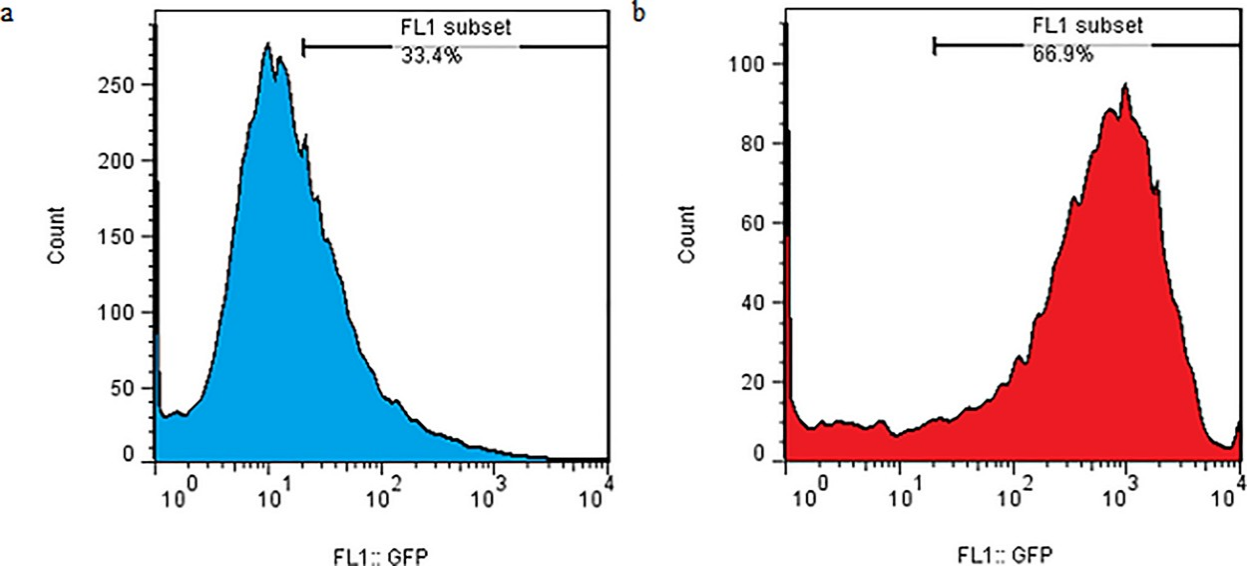
Effect of uniaxial cyclic stretch on TC-1 transfection efficiency by flow cytometry (loading time: 15 min, frequency: 0.5 Hz, and strain: 5%). (a) without reagent (b) with reagent.

**Fig 6:**
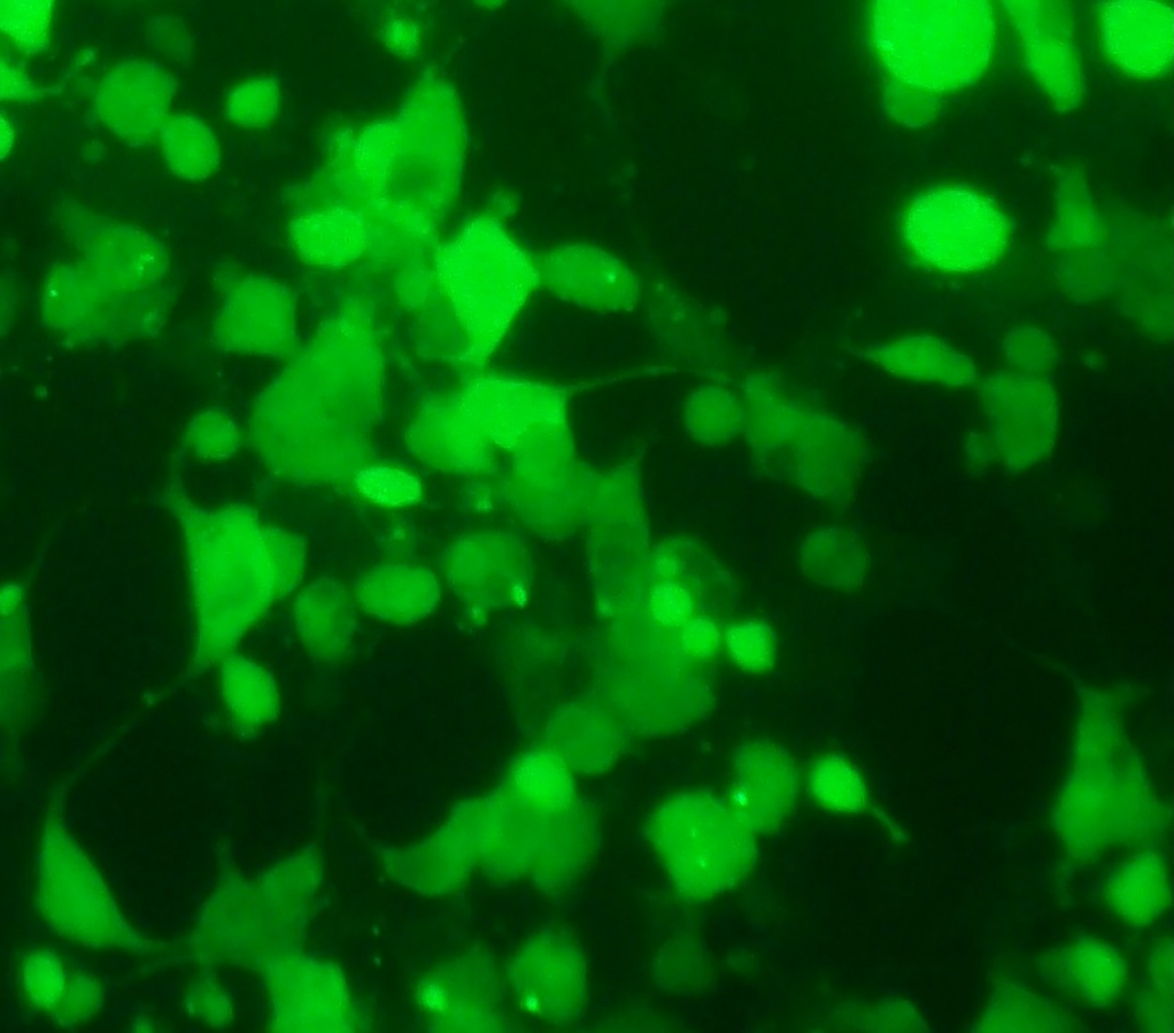
The transfection efficiency of pEGFP-N1 in TC-1 related to 15 min loading time for simultaneous use of mechanical loading of uniaxial cyclic stretch and chemical reagent by fluorescent microscopy.

The combined effect of mechanical loading and TurboFect transfection reagent on the gene transfection in the TC1 cells were investigated. In this case, at first, the membrane containing the cells was loaded and then the transfection operation was performed using a plasmid DNA with TurboFect transfection reagent. This test was done under optimal conditions determined in section 3.2 (strain rate = 0.5%, frequency = 0.5 Hz). The survey parameter was loading time for the simultaneous use of mechanical loading of uniaxial cyclic stretch and chemical reagent. The results were shown in Fig 4. This test also indicates that the maximum transfection was related to 15 min loading time (63.07 ± 5.24%). It can also be concluded that the chemical reagent increased the efficiency of transfection by mechanical loading. Also, the results of flow cytometry were indicated in Fig 5 in two states. In the first state, the cell is affected by uniaxial cyclic stretch, and in the second state, the TurboFect transfection reagent was also employed in addition to uniaxial cyclic stretch. The statistical results showed that the application of an effective parametric the TurboFect transfection reagent was to assist in transfection under uniaxial cyclic stretch. Fig 6 illustrated the image of a fluorescent microscope to investigate the efficiency of transfection for 15 min loading time and frequency 0.5 Hz for simultaneous use of mechanical loading of uniaxial cyclic stretch and the TurboFect transfection reagent.

### 3.4 The effect of equiaxial cyclic stretch and the simultaneous use equiaxial cyclic stretch and TurboFect transfection reagent

**Fig 7:**
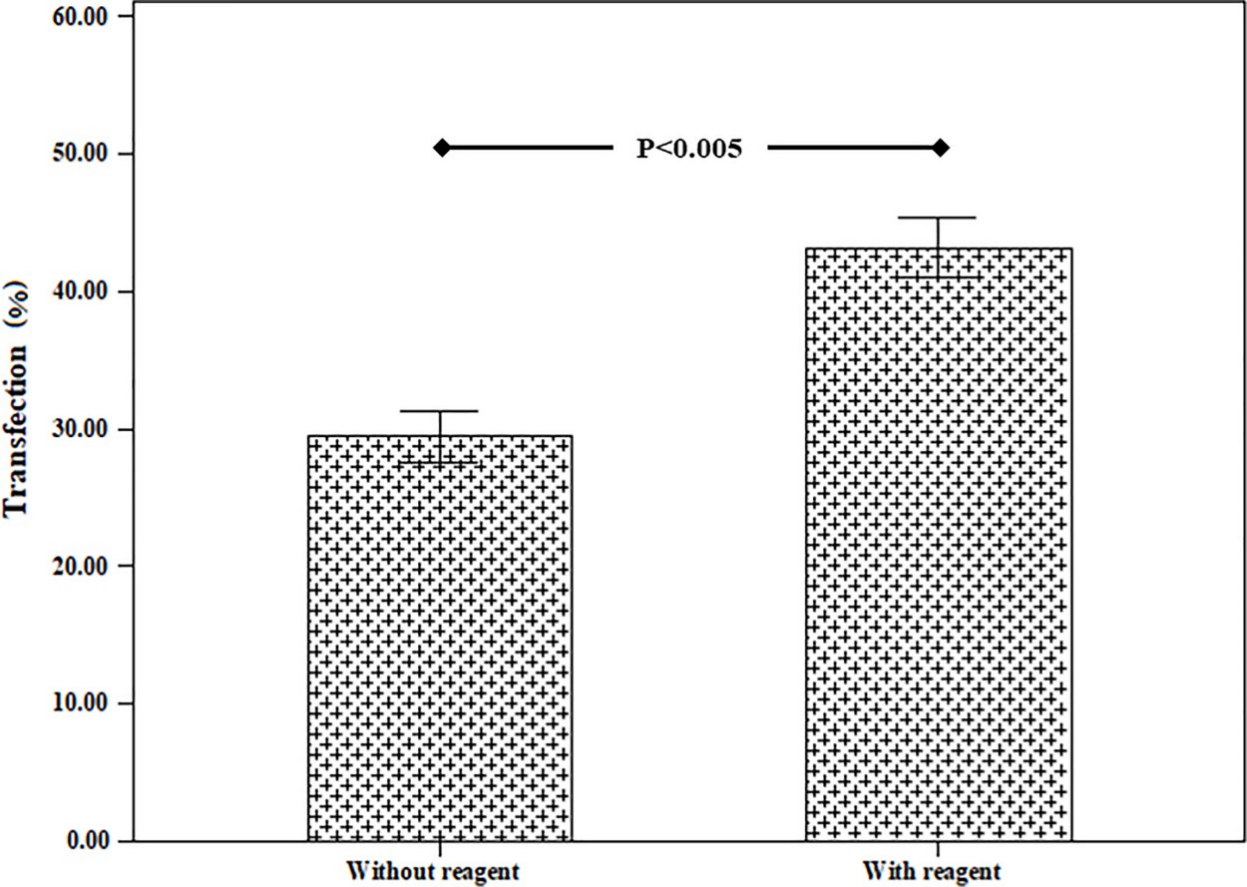
Comparison of efficiency of equiaxial cyclic stretch transfection methods with and without reagent (frequency 0.5 Hz, strain rate of 5% and loading time of 15 minutes).

**Fig 8:**
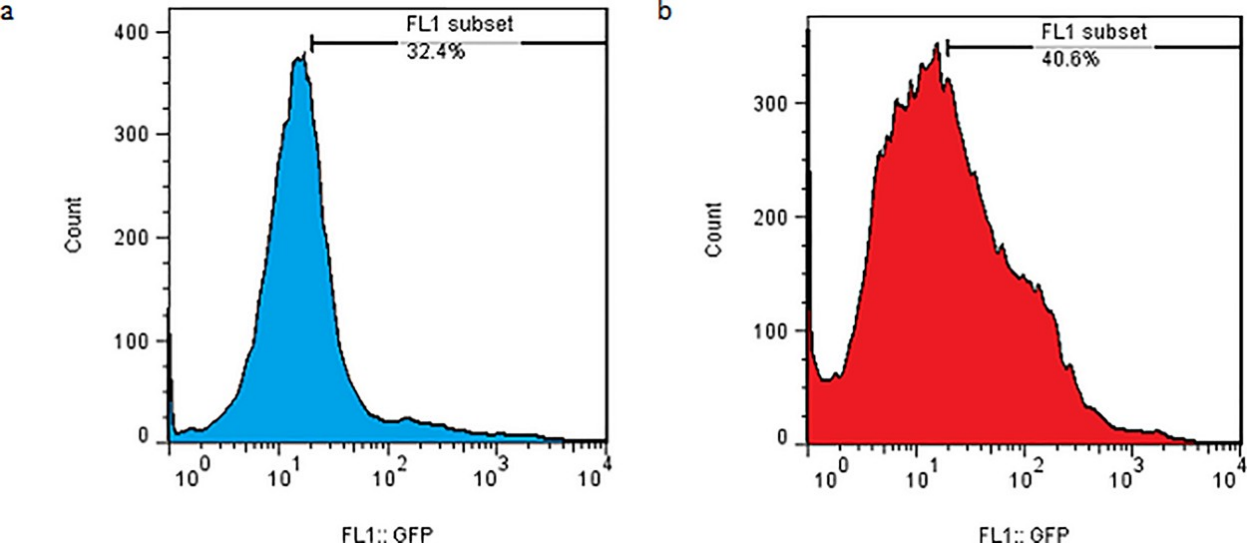
Flow cytometry analysis for equiaxial cyclic stretch transfection of TC-1 (loading time: 15 min, frequency: 0.5 Hz, and strain: 5%). (a) without reagent (b) with reagent.

The effect of equiaxial cyclic stretch and the simultaneous effect of mechanical loading of equiaxial cyclic stretch and TurboFect transfection reagent as a gene transfection factor have been investigated (Fig 7). In this test, according to the loading conditions of the previous section, loading parameters were selected. The mechanical loading conditions were frequency 0.5 Hz, strain rate of 5% and loading time of 15 minutes. The results of this section showed that the transfection percentage due to equiaxial cyclic stretch was 29.43 ± 3.17%. The combination of equiaxial cyclic stretch loading and TurboFect transfection reagent increased the transfection efficiency to 43.17 ± 3.75%. Fig 8 revealed the results of flow cytometry derived from the transfection of equiaxial cyclic strain for two states of use and no use of the TurboFect transfection reagent. This figure also demonstrated that using the TurboFect transfection reagent increases the transfection efficiency.

### 3.5 The effect of shear stress and the simultaneous use of shear stress and TurboFect transfection reagent

**Fig 9:**
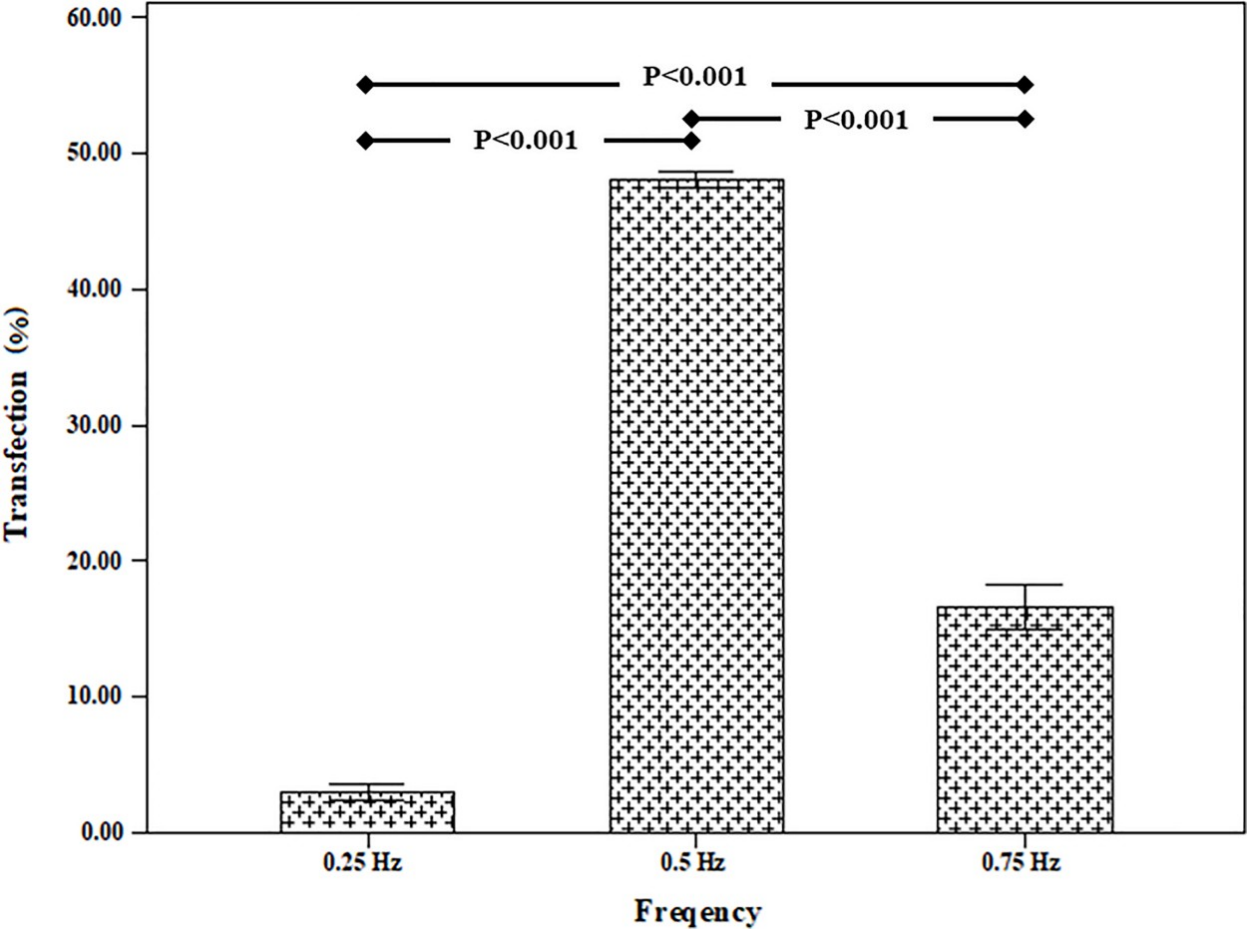
Effect of simultaneous use of mechanical loading of shear stress and chemical reagent (TurboFect Transfection Reagent) for various frequency (loading time = 15 min).

**Fig 10:**
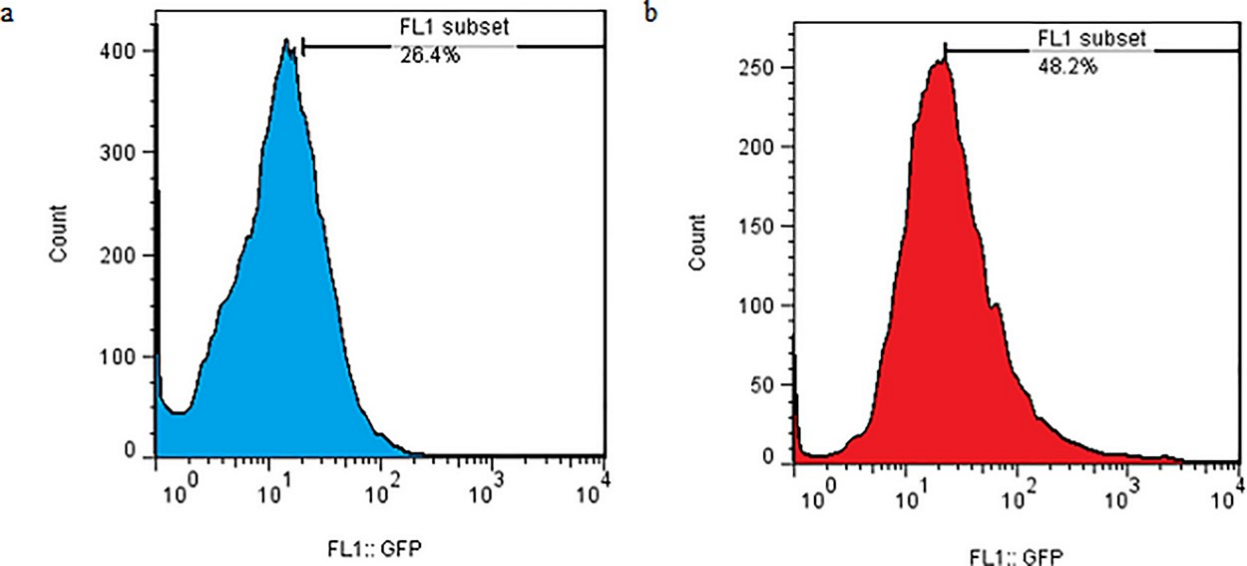
Effect of shear stress on TC-1 transfection efficiency by flow cytometry (loading time: 15 min, and frequency: 0.5 Hz). (a) without reagent (b) with reagent.

The effect of shear stress loading was investigated for transferring the gene into the cultured cell on the silicon membrane. Fig 9 revealed the effect of frequency on the transfection efficiency for 15 minute loading time. The results demonstrated that the frequency equal to 0.5 Hz experienced a significant difference compared to other two frequency groups. The flow cytometry results at this frequency for the two states of use or no use of the TurboFect transfection reagent had been listed in Fig 10. The statistical results indicated that the use of the turbofect transfection reagent was effective in increasing the transfection efficiency caused by shear stress.

The results showed that shear stress with a loading time of 15 minutes and frequency 0.5 Hz caused gene transfection of 26±2.23%. Moreover, the simultaneous effect of mechanical loading of the shear stress and the chemical reagent caused a gene transfection of up to 48.1±0.96%.

## Discussion

This paper investigated the transfection efficiency of pEGFP-N1 in TC1 cell line. This cell line has a low efficiency of gene transfection by commercial transfection reagent. Shahbazi et al. [43] showed the pEGFP-N1 and pEGFP-E7 delivery were detected in approximately 99.1% and 80.63% of HEK-293T cells, 5.04% and 4.47% of TC-1 cells, and 6.66% and 5.95% of A549 cells treated with TurboFect, respectively. These results indicated that TurboFect produced more effective transfection in the non-cancerous cells compared to cancerous cell lines [43]. Transfection of cancer cell line possesses many applications including *in vivo* medical imaging and therapeutic strategy for cancer [44–46]. Therefore, DNA transfection efficiency in the cancer cell line is facing a challenge. Molecular dynamics research suggests that exerting mechanical load was accompanied by the formation of cavities in the cell membrane, which could cause cell permeability [43]. Akimov *et al*. [47] indicated the effective mechanical force was driving changes of the pore radius. Akimov *et al*. [48] in another study, considered the process of pore formation under the external stress. The waiting time to pore formation proved a nonmonotonous function of the lateral tension, dropping from infinity at zero tension to a minimum at the tension of several milli-newtons per meter. So, we could conclude mechanical stimuli were a strategy for the challenge of transfection of cancer cells. A study based mechanical loading on cell indicated that a prototype of a microfluidic-based jet injector designed to deliver macromolecules inside a cell by piercing the cell wall with a picoliter jet [9]. The limitation of this study was transfection only a small number of cells. Sharei et al. [49] provided a microfluidic system for transfection macromolecules based on the immediate deformation of cell. This system transmits macromolecules into the cell by compression. The considered system was capable of transfection 20,000 cell/sec up to 1,000,000 cells until failure of channels of this microfluidic system by clogging. It should be noted that 1,000,000 cells with each platform could be referred to as a limitation of microfluidic systems, but the system provided in the present study was by cell culture on a silicone membrane and loading of this membrane. The membrane is capable of bearing loads in this range and almost an unlimited number can be used for transfection. Also, this system can simulate different stress fields on cells.

In our study, the effects of mechanical loading on TC1 cells for transfection of the plasmid harboring GFP gene were investigated. In our study for mechanical loading, uniaxial cyclic stretch bioreactor, equiaxial cyclic stretch, and shear stress bioreactors in the Pasteur Institute of Iran were used. For chemical DNA transfection, the TurboFect transfection reagent had been used. The results of this study showed that mechanical loading alone could be a factor for gene transfection into the cell. Suitable loading conditions for gene transfection using uniaxial cyclic stretch bioreactor were loading time of 15 minutes with the frequency of 0.5 Hz and strain percentage 5%. In this loading condition, the transfection efficiency was 30.20 ± 3.02%. In this test, only mechanical stimuli acted on the cell. Therefore, it could be concluded that the mechanical loading, alone, was a great transfection factor. In the next experiment, under conditions (strain rate = 5%, loading time = 15 min and frequency = 0.5Hz), equiaxial cyclic stretch was applied on TC-1 cells. The transfection efficiency for this test was 29.43 ± 3.17%. Also, this test confirmed that the mechanical loading was an efficient approach for delivery of DNA into the hard-transfected cell. The simultaneous use of the TurboFect transfection reagent and mechanical loading increased the transfection efficiency up to 63.06 ± 5.24% and 43.17 ± 3.75% for uniaxial cyclic stretch and equiaxial cyclic stretch loadings, respectively. Our data indicated that the chemical transfection enhanced the efficiency of mechanical loading. On the other hand, given that the TurboFect transfection reagent alone could not be effective for DNA transfection into TC-1 cells, we could understand that the mechanical forces caused the transient pores in phospholipid membrane and this transient pores helped to increase the efficacy of TurboFec transfection reagent. In the final experiment, the effect of shear stress on transfection of TC1 was examined. The results of this study showed that shear stress could also be a factor in the gene transfer into the TC-1 cell.

In conclusion, this study introduced that mechanical loading alone could be a factor in transfection of the gene into the cell. The simultaneous use of mechanical loading and commercial chemical reagent increased the transfection efficiency. This method can be used for cells with low transfection efficiency like cancer cell line and immune cells [50]. Transfection of immune cells like dendritic cells (DCs) with tumor antigens can stimulate potent antitumor immunity in tumor‐bearing mice [51–55].

## Supporting information

S1 Fig The transfection efficiency of pEGFP-N1 in TC-1 loading time for use of mechanical loading of uniaxial cyclic stretch under frequency 0.5 Hz. (a) Control TC-1. (b) 3% strain, 60 min loading. (c) 5% strain, 60 min loading. (d) 10% strain, 60 min loading. (e) 5% strain, 180 min loading. (f) 5% strain, 15 min loading. (g) 5% strain, 5 min loading. (h) 5% strain, 60 min loading and use of chemical factor. (i) 5% strain, 15 min loading and use of chemical factor. (j) 5% strain, 5 min loading and use of chemical factor.
(DOCX)
