# Supplementary material for: Enhanced gene delivery in tumor cells using chemical carriers and mechanical loadings
Source: PLoS One. 2018 Dec 28;13(12):e0209199. doi: 10.1371/journal.pone.0209199 (PMC6310266; doi:10.1371/journal.pone.0209199)
Supplement: S1 Fig — (a) Control TC-1. (b) 3% strain, 60 min loading. (c) 5% strain, 60 min loading. (d) 10% strain, 60 min loading. (e) 5% strain, 180 min loading. (f) 5% strain, 15 min loading. (g) 5% strain, 5 min loading. (h) 5% strain, 60 min loading and use of chemical factor. (i) 5% strain, 15 min loading and use of chemical factor. (j) 5% strain, 5 min loading and use of chemical factor. (DOCX) [file pone.0209199.s001.docx]

| 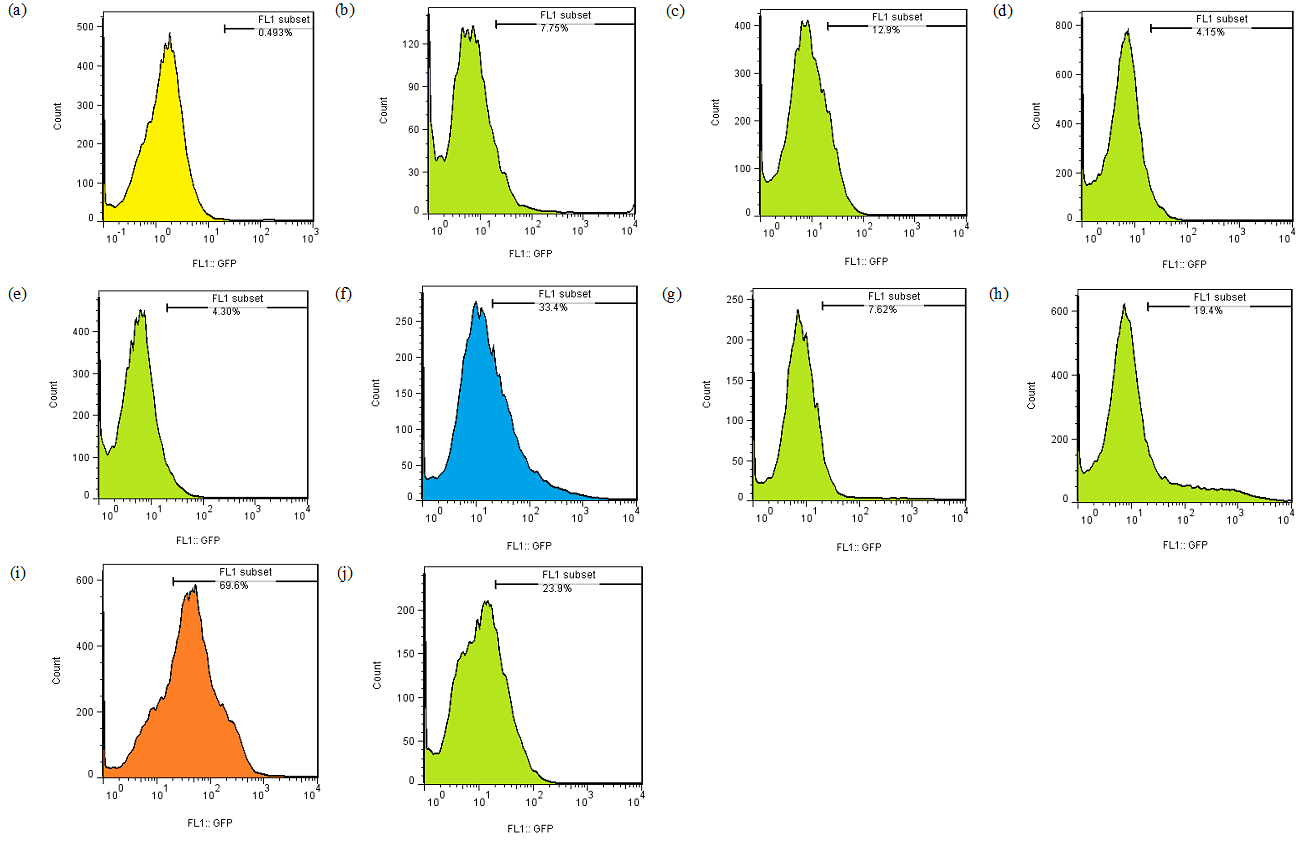 |
| --- |
| **S1 Fig. The transfection efficiency of pEGFP-N1 in TC-1 loading time for use of mechanical loading of uniaxial cyclic stretch under frequency 0.5 Hz. (a) Control TC-1. (b) 3% strain, 60 min loading. (c) 5% strain, 60 min loading. (d) 10% strain, 60 min loading. (e) 5% strain, 180 min loading. (f) 5% strain, 15 min loading. (g) 5% strain, 5 min loading. (h) 5% strain, 60 min loading and use of chemical factor. (i) 5% strain, 15 min loading and use of chemical factor. (j) 5% strain, 5 min loading and use of chemical factor.** |
